# Supplementary material for: Modeling the longitudinal changes of ancestry diversity in the Million Veteran Program
Source: Hum Genomics. 2023 Jun 2;17:46. doi: 10.1186/s40246-023-00487-3 (PMC10239111; doi:10.1186/s40246-023-00487-3)
Supplement: Supplementary file 2 — Additional file 2: Figure S1. Height across birth cohorts. Small changes in height exist across birth cohorts in the MVP. The change in height between two birth cohorts correlates with change in mean GBR ancestry proportion. Each data point is a pairwise comparison of GBR ancestry proportion within each HARE superpopulation. EUR European, AFR African, EAS East Asian, HIS Hispanic. Figure S2. SNP-heritability comparisons across GWAS of height performed in HARE superpopulations and populations assigned using a high-resolution ancestry reference panel composed of 1000 Genomes Project plus Human Genome Diversity Project individuals. Each GWAS was performed in unrelated participants of the indicated ancestry with age, sex, and 10 within-population principal components as covariates. EUR European, AFR African, EAS East Asian, HIS Hispanic. Figure S3. Attenuation ratio comparisons across GWAS of height performed in HARE superpopulations and populations assigned using a high-resolution ancestry reference panel composed of 1000 Genomes Project plus Human Genome Diversity Project individuals. Each GWAS was performed in unrelated participants of the indicated ancestry with age, sex, and 10 withing-population principal components as covariates. EUR European, AFR African, EAS East Asian, HIS Hispanic. [file 40246_2023_487_MOESM2_ESM.docx]

**Supplementary Figures**


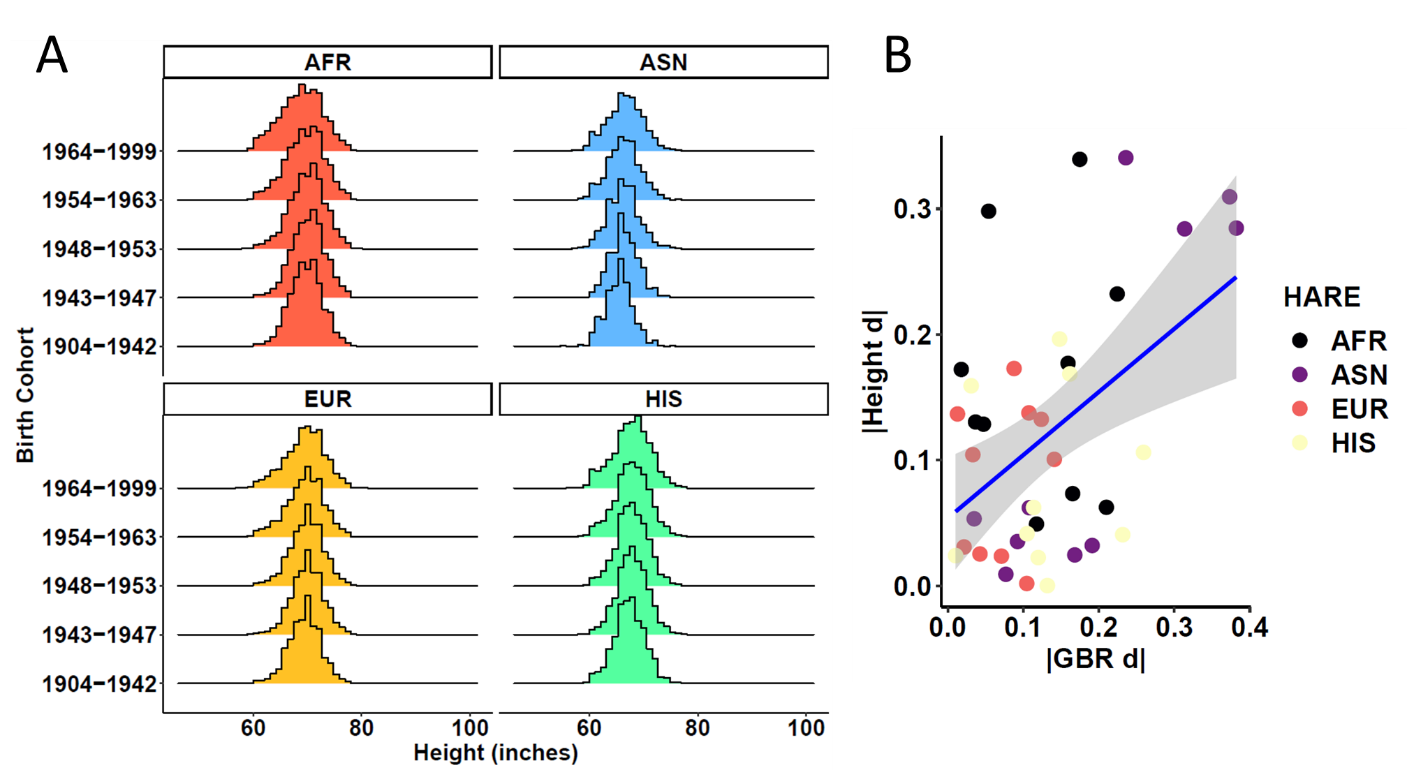


Figure S1. Height across birth cohorts. (A) small changes in height exist across birth cohorts in the MVP. (B) The change in height between two birth cohorts correlates with change in mean GBR ancestry proportion. Each data point is a pairwise comparison of GBR ancestry proportion within each HARE superpopulation. Abbreviations: European (EUR); African (AFR); East Asian (EAS); Hispanic (HIS).


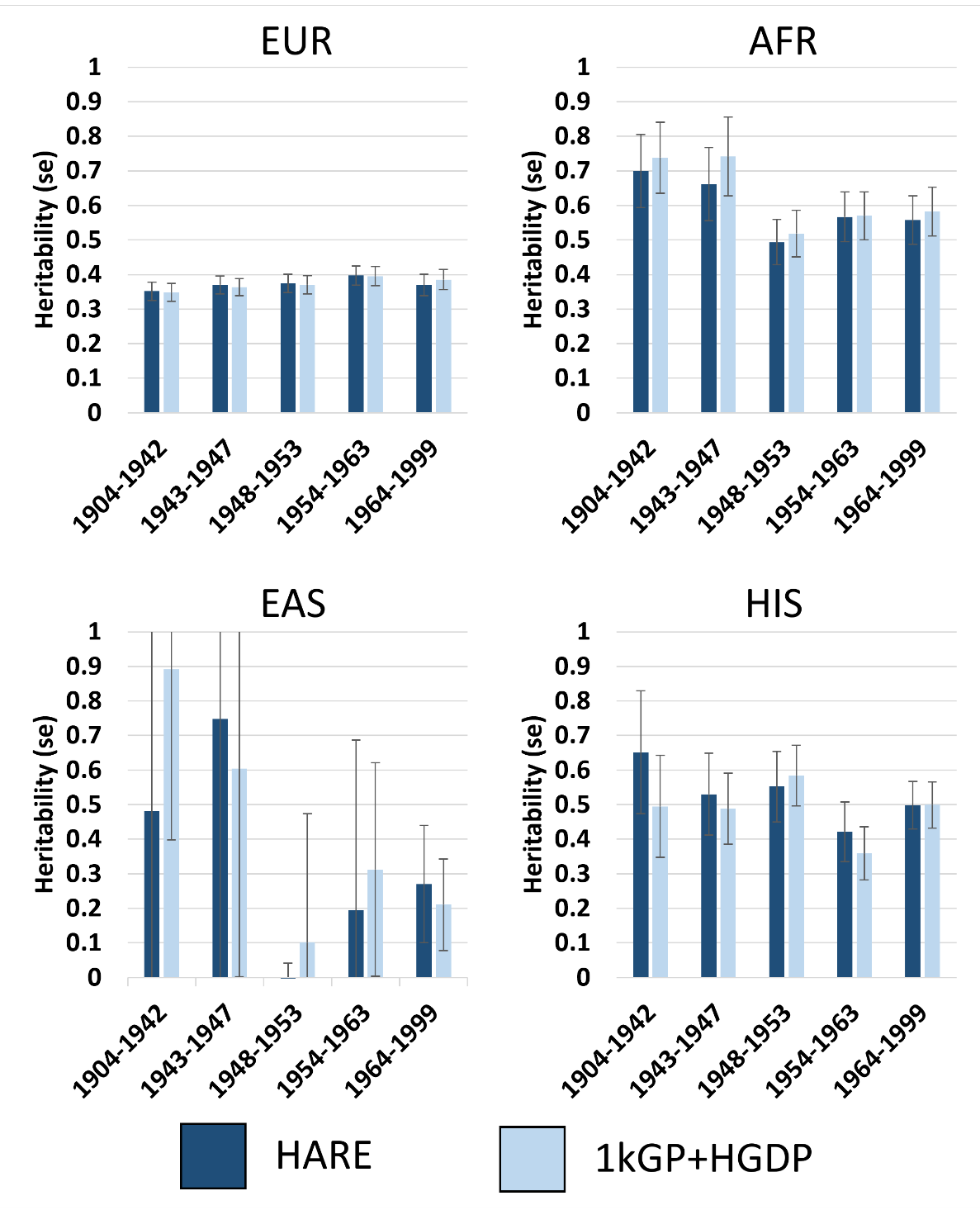


Figure S2. SNP-heritability (h^2^) comparisons across GWAS of height performed in HARE superpopulations and populations assigned using a high-resolution ancestry reference panel composed of 1000 Genomes Project plus Human Genome Diversity Project individuals (1kGP+HGDP). Each GWAS was performed in unrelated participants of the indicated ancestry with age, sex, and 10 withing-population principal components as covariates. Abbreviations: European (EUR); African (AFR); East Asian (EAS); Hispanic (HIS).


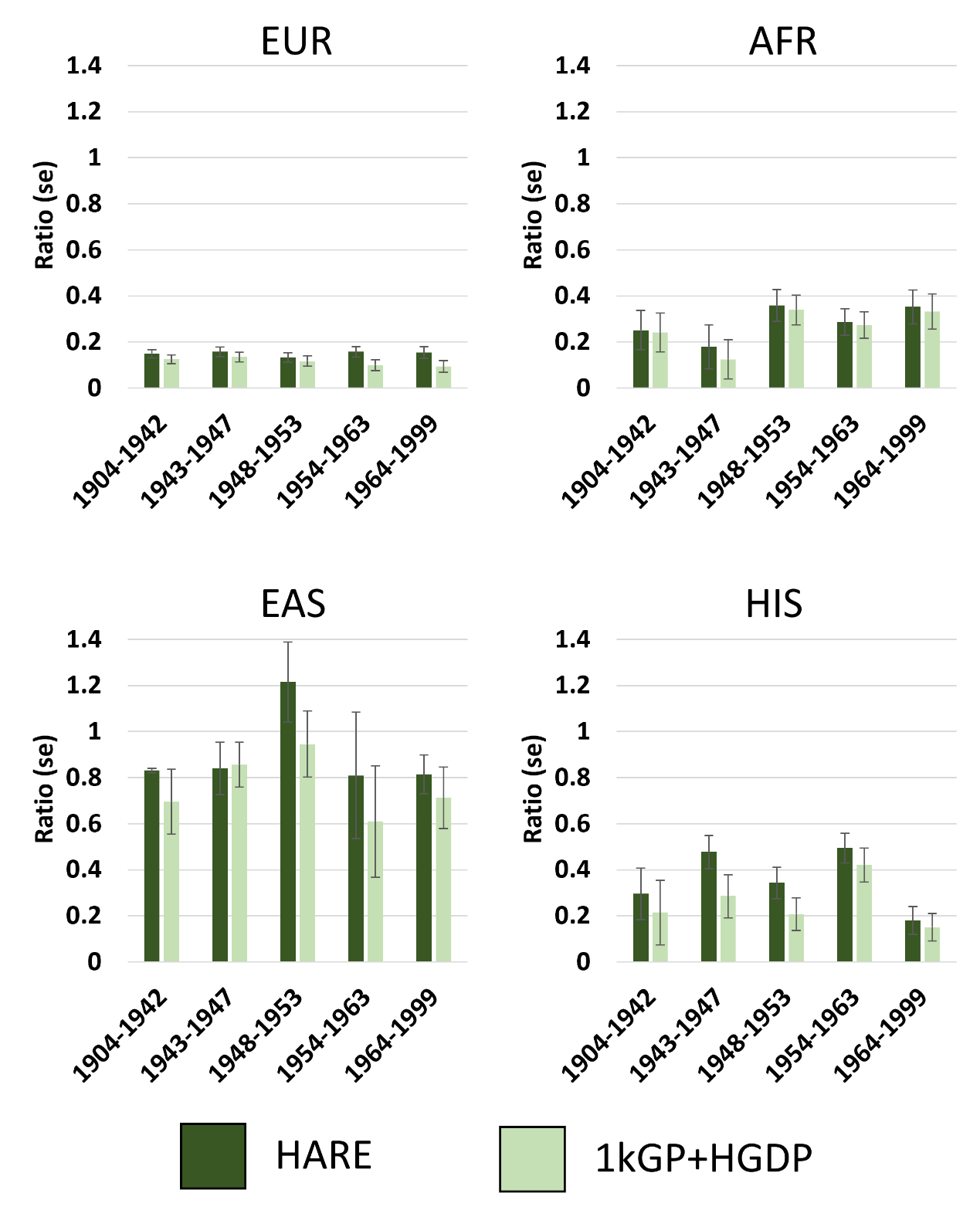


Figure S3. Attenuation ratio comparisons across GWAS of height performed in HARE superpopulations and populations assigned using a high-resolution ancestry reference panel composed of 1000 Genomes Project plus Human Genome Diversity Project individuals (1kGP+HGDP). Each GWAS was performed in unrelated participants of the indicated ancestry with age, sex, and 10 withing-population principal components as covariates. Abbreviations: European (EUR); African (AFR); East Asian (EAS); Hispanic (HIS).

**Supplementary Tables**

Table S1. Patterns of service era per birth cohort ("Era") and across all MVP participants stratified by sex and HARE superpopulations. Each row represents a distinct pattern of service across nine service eras; the frequency of each is calculated by birth cohort and for all MVP participants. Service patterns with less than 11 participants were omitted to preserve data privacy of the participant so HARE total population sample sizes are slightly lower than those reported in Table 1.

Table S2. Sample size per birth cohort derived from cumulative distribution function of year of birth.

Table S3. Mean ancestry proportion of five 1kGP reference populations in all birth cohorts and HARE superpopulations. Two-sided Z-tests were used to compare the statistical difference in means between groups and the corresponding p-values reflect this difference. Standardized mean differences (Cohen's d) reflects the magnitude of effect size difference between two groups.

Table S4. Comparison of height across birth cohorts in each MVP HARE superpopulations.

Table S5. Metrics for GWAS of height in each ancestry per birth cohort using both methods of population assignment. Heritability (h2), LDSC intercepts, and attenuation ratios were compared across birth cohorts, within each method, using two-sided Z-tests. Multiple testing correction was applied using a false discovery rate of 5%; differences surviving multiple testing correction are highlighted in yellow.

Table S6. Metrics for GWAS of height compared across method used to define superpopulations. Two-sided Z-tests were used to compare heritability (h2), LDSC intercepts, and attenuation ratios between HARE and 1kGP+HGDP superpopulation assignments. Multiple testing correction was applied using a false discovery rate of 5%.
